# Validating enzyme immunoassays for non-invasive reproductive hormone monitoring in Temminck’s pangolin

**DOI:** 10.1093/conphys/coae079

**Published:** 2024-11-14

**Authors:** Juan Scheun, Andre Ganswindt, Raymond Jansen, Kim Labuschagne

**Affiliations:** Department Nature Conservation, Tshwane University of Technology, Staatsartillerie Rd, Pretoria West, Pretoria 0183, South Africa; Faculty of Natural and Agricultural Sciences, Mammal Research Institute, University of Pretoria, Lynnwood Rd, Hatfield, Pretoria 0002, South Africa; Faculty of Natural and Agricultural Sciences, Mammal Research Institute, University of Pretoria, Lynnwood Rd, Hatfield, Pretoria 0002, South Africa; Department of Environmental, Water and Earth Science, Tshwane University of Technology, Staatsartillerie Rd, Pretoria West, Pretoria 0183, South Africa; IUCN SSC Pangolin Specialist Group, c/o Zoological Society of London, London NW1 4RY, UK; SANBI Wildlife Biobank, South African National Biodiversity Institute, 232 Boom St, Daspoort 319-Jr, Pretoria 0001, South Africa

**Keywords:** Conservation, endocrine monitoring, non-invasive, pangolin, reproduction

## Abstract

Gonadal hormones play a central role in reproductive function and success. As such, quantifying reproductive hormones non-invasively in threatened, vulnerable and endangered wildlife species offers an ideal tool for assessing general and individual reproductive patterns *in situ*. Whilst the use of faeces as a hormone matrix is often preferred in these cases, the required enzyme immunoassays (EIAs) for measuring faecal androgen (fAM), oestrogen (fEM) and progestagen metabolite (fPM) concentrations must first be validated if a species gets investigated for the first time to ensure biologically relevant patterns can be observed. In this study we aimed to biologically validate the EIAs for monitoring fAM, fEM and fPM concentrations in Temminck’s pangolin, *Smutsia temminckii*. Hormone metabolite concentrations derived from each EIA tested were compared between different age and sex classes. An epiandrosterone EIA effectively measured androgen levels in males, distinguishing between adult and juvenile individuals, as well as both female age classes. Similarly, the tested oestrogen EIA successfully distinguished between adult and juvenile female fEM concentrations, and both tested progestagen EIAs demonstrated adequate differences between fPM concentrations of adult and juvenile females. The now-validated EIAs offer robust tools for a non-invasive monitoring of reproductive activity in Temminck’s pangolin. The development of such techniques will allow researchers to assess reproductive hormone patterns of the species *in situ*, whilst also paving the way for further studies in this field. Despite the small sample size due to the species’ conservation status, the study provides a foundation for future research using a robust, validated, non-invasive monitoring tool. The latter can now be implemented in long-term monitoring with larger sample sizes to yield more comprehensive data, aiding in the conservation of Temminck’s pangolin.

## Introduction

Hormone monitoring has emerged as an important technique in the field of wildlife conservation, offering invaluable insights into stress and reproductive physiology in wildlife species (Möstl and Palme, 2002; Hodges *et al.,* 2010; Sheriff *et al.,* 2011). This has enabled researchers to gain a better understanding of animal behaviour, reproductive status, population dynamics, survival and health (Lipschitz, 1997; Schwarzenberger and Brown, 2013; Cain and Cidlowski, 2017). In terms of reproduction, it is the hypothalamic–pituitary–gonadal (HPG) axis that is responsible for initiating, regulating and coordinating most of the reproductive functions in vertebrate species (Saltzman *et al.,* 2011). In many free-roaming species a change in specific seasonal parameters, such as temperature or rainfall, act as indicators of the approaching breeding season and triggers breeding activity, both on a behavioural and physiological level (Bronson and Heideman, 1994; Bronson, 2009). Monitoring the reproductive physiology of a species enables researchers to decipher the complex mechanisms that underlie courtship, mating and other reproductive behaviours (Christensen *et al.,* 2012; Watts, 2020). Here, researchers often monitor the so-called ‘male (androgen) and female (oestrogen and progestagen)’ reproductive hormones, which are important for the development of secondary sexual characteristics, the activation of reproductive behaviours, sperm production in males as well as cyclicity and pregnancy maintenance in females (Beehner *et al.,* 2006a; Beehner *et al.,* 2006b; Muller, 2017).

Although blood collection and analyses are often regarded as the gold standard of reproductive endocrine monitoring, providing real-time gonadal hormone concentrations (Kumar and Umapathy, 2019), the method requires researchers to trap and restrain individuals for extended periods of time. Not only can this result in animal and/or researcher injuries, but prolonged stressful events to the individual can also impact on reproductive processes and foetal development, should such events occur regularly (Wu *et al.,* 2020; Fogel *et al.,* 2023). Due to the invasive nature of endocrine monitoring using blood, this technique has fallen out of favour with many members of the research community, especially those working on threatened species (Kersey and Dehnhard, 2014). This is especially relevant to pangolin species, who roll into a tight ball as part of its defensive behaviour; as a result of this, individuals must be anaesthetised (Hooijberg *et al.,* 2021), which is not only invasive but leads to extended handling and recovery times. Considering these difficulties, many researchers have opted for examining endocrine patterns using non-invasive monitoring techniques. Reproductive steroid hormones are predominantly secreted by the HPG axis into the bloodstream and are metabolized by the liver and excreted as conjugates in bile and ultimately faeces (Grow, 2002; Rhyu and Yu, 2021). As such, monitoring steroid hormone metabolite concentrations in faeces can provide a robust proxy of gonadal function in an organism (Schwarzenberger, 2007; Hodges *et al.,* 2010).

Despite the numerous advantages of using non-invasive endocrine metabolite monitoring, hormone secretion, metabolism and excretion are species- and sex-specific (Goymann, 2005; Goymann, 2012). As a result of this, any enzyme immunoassay (EIA) hormone matrix combination, employed to monitor the reproductive physiology in a species for the first time, must be validated to ensure a reliable quantification of respective male and female reproductive biomarkers (Touma and Palme, 2005; Palme, 2019). Such EIA validations can be conducted via physiological or biological means. Physiological validations include the injection of a substance to hyperactivate the HPG axis; faecal samples are collected prior to and following the injection and analysed with specifically chosen EIAs for each hormone class in order to determine which assays can reliably detect biologically relevant changes in faecal hormone metabolite concentrations (Pribbenow *et al.,* 2016; Scheun *et al.,* 2018). However, due to its invasive nature, it is not always possible to implement a physiological validation, especially in threatened species (Touma and Palme, 2005; Eckardt *et al.,* 2016). In these instances, a biological validation can be used to successfully validate an appropriate EIA system for measuring the reproductive physiology in a species. Here, sex- and age-specific differences in hormone metabolite concentrations can be used to validate EIAs for monitoring reproductive endocrine patterns in a species (Wielebnowski and Watters, 2007). Generally, adult and pregnant females have higher faecal oestrogen (fEM) and progestagen metabolite (fPM) concentrations than juvenile and adult non-pregnant females or males of any age class; similarly, adult males have higher faecal androgen metabolite (fAM) concentrations than juvenile males or any female age class (Pineda-Galindo *et al.,* 2017; Scheun *et al.,* 2017).

Once respective monitoring systems are in place, they can be used to enhance our understanding and drive further research into the reproductive biology of the species *in situ,* specifically by determining the reproductive status and sex determination in the field (Tubbs *et al.,* 2014; Scheun *et al.,* 2016; Brown, 2018). Importantly, endocrine metabolite monitoring can be used as a key indicator of the ability of a species to reproduce and/or adapt to changing environmental conditions. Non-invasive endocrine monitoring offers an easy-to-use technique to assist in the management and conservation of endangered and vulnerable wildlif species.

Temminck’s pangolin (*Smutsia temminckii*), is listed as globally vulnerable by the International Union for Conservation of Nature (Pietersen *et al.,* 2019). The species is facing several anthropogenically driven threats, including the illegal wildlife and medicine trade, habitat loss, electrical fences and overutilization (Pietersen *et al.,* 2014a). In addition to these threats, individuals retrieved from the wildlife trade go through prolonged rehabilitation and release processes (Wright and Jimerson, 2020). Despite the often-prolonged rehabilitation processes, no information has been gathered on reproductive physiology in the species. Rehabilitation efforts, coupled with direct monitoring of populations, are critical to understanding and enhancing their survival prospects (Challender *et al.*, 2019). These initiatives help bridge gaps in our understanding of their ecological needs and reproductive cycles, crucial for formulating effective conservation strategies such as conducting a Population Viability Analysis. To assist in this regard, it is important that new methods for determining the reproductive status sex determination be developed. As such, the aim of this study was to validate EIAs for a reliable monitoring of fAM, fEM and fPM concentrations in Temminck’s pangolin.

## Materials and Methods

### Study animals

Due to the conservation status and pattern of population decline throughout the species’ distribution range, it is not possible to capture and/or hold Temminck’s pangolin for extended periods of time in order to conduct research. Furthermore, as pangolins are highly susceptible to stress (Wicker *et al.,* 2020), long-term housing for research purposes may lead to a decrease in health and reproductive function in captive individuals (Dobson and Smith, 2000).

Owing to their low population densities, low detection rates and the inability to maintain them in full captivity for any length of time, there is a dearth in physiological data. As such, we depended on opportunistic samples collected at various facilities involved in the rehabilitation and release of confiscated individuals. Though this provided us with an opportunity to collect faecal samples from several male and female adult and juvenile individuals, it should be noted that the rehabilitation process puts an emphasis on limiting any human–pangolin interaction. As such, long-term sample collection from individuals was not possible. A trained veterinarian ensured individual health and welfare of all individuals used in this study. In addition to this, pregnancy status was also determined by the veterinarian through abdominal palpation and abdominal sonar.

#### Participating facilities and animal care

The Johannesburg Wildlife Veterinary Hospital, Loskop Dam Nature Reserve and The Munywana Conservancy all contributed samples to this study. Temminck’s pangolins are not held at these facilities but at an offsite location for the safety of the individuals and staff members. Pangolin are kept in large individual pens (>8 m^2^) and all care, including daily walks ranging from 2 to 6 h per individual, as well as feeding are conducted by facility staff as per established pangolin rehabilitation and care protocols.

#### Ethical clearance

The study was performed with the approval of the University of South Africa’s Animal Research Ethics Committee (2020/CAES_AREC/109).

### Biological validation process

Due to restrictions on retaining individuals for research purposes, and the impracticality of conducting physiological validations, we chose to employ biological validations for determining the most suitable EIAs to monitor reproductive physiology in Temminck’s pangolin.

To biologically validate the most appropriate EIAs for monitoring fEM, fPM and fAM concentrations in Temminck’s pangolin, we compared the respective median metabolite concentrations between adult (>6 kg) and juvenile (<6 kg) male and female individuals (Pietersen *et al.,* 2014b). A total of three samples were collected from each of the 10 study animals, resulting in a total of 30 samples (five females (two adults, three juveniles) and five males (three adults and two juveniles)). Sample collection occurred over a year period. As Temminck’s pangolin are not seasonal breeders (Pietersen *et al.,* 2014b), this span should not influence observed endocrine patterns over this period. A single faecal sample was collected from a pregnant female (as determined by the veterinarian). All fresh faecal samples were placed into individual sample collection bags, sealed and stored at −20°C until transported to the SANBI Biobank.

### Faecal sample extraction and analysis

All frozen faecal samples were extracted at the SANBI Wildlife Biobank following the methods used by Long *et al.* (2021). Faecal samples were lyophilized, pulverized and sieved through a thin mesh to remove any non-faecal matter that might be present. Subsequently, 1.5 ml of 80% ethanol was added to 0.050–0.055 g of faecal powder and vortexed for 15 min, before centrifuging the samples at 1500 × *g* for 10 min. The supernatant was then transferred to clean, clearly marked 2.5-ml microcentrifuge tubes, sealed and stored at −20°C until EIA analyses at the Endocrine Research Laboratory (ERL), University of Pretoria, South Africa. EIA analyses occurred within 3 months post-extraction.

All faecal extracts were measured for immunoreactive fEM, fPM and fAM concentrations, using an (i) oestrogen EIA, two progestagen EIAs ((ii) progesterone and (iii) 5α-progesterone) and two androgen EIAs ((iv) epiandrosterone, (v) testosterone). Details of the assays, including cross-reactivities of the antibodies, are described by Palme and Möstl (1993) for oestrogen, testosterone and epiandrosterone, Schwarzenberger *et al.* (1996b) for progesterone and Dehnhard *et al.* (2010) for 5α-progesterone. Antibody, conjugate and standard for the 5α-Progesterone EIA were purchased from Leibniz-Institute for Zoo Biology and Wildlife Research, PF 601103, D-10252 Berlin, Germany. Antibodies, conjugates and standards for the remaining EIAs (Oestrogen, Testosterone, Epiandrosterone and Progesterone) were purchased from the Department of Biomedical Sciences/Physiology, University of Veterinary Medicine, Veterinärplatz 1, A-1210 Vienna, Austria (contact: Associate Prof. Dr R. Palme, Rupert.Palme@vetmeduni.ac.at). Information on the conjugates is available in the cited publications. Plates were coated (Corning Product Number 9018) with AR-IgG (Arbor Assays, USA) using a standard procedure. Serial dilutions of extracted samples gave displacement curves that were parallel to the respective standard curves in all assays (Table 1). The intra- and inter-assay coefficient of variance (CV), determined by repeated measurements of high- and low-value quality controls, as well as assay sensitivities are shown in Table 1. Concentrations measurements in the faecal extracts were carried out in different dilutions, all within the parallel ranges of the respective EIAs. Diluted Standards were used as quality controls, at 20 and 80% binding for the respective EIAs. The standards can be provided by the suppliers of the antibodies and conjugates. As the faecal extract dilutions could be increased, no maximum detection value was reported.

**Table 1:** The intra- and inter-assay CV, assay sensitivity, as well as parallelism test results and dilution ranges for the faecal oestrogen, progestogen and androgen metabolite enzyme immunoassays

Enzyme immunoassay	Intra-assay CV	Inter-assay CV	Assay sensitivity ng/g faecal dry mass	Parallelism test	Dilution ranges used in the parallelism test
Testosterone	6.58 and 7.59%	8.02 and 14.93%	0.4	<4%	1/20–1/8000
Epiandrosterone	4.96 and 5.09%	6.89 and 9.67%	2.4	<4%	1/50–1/8000
Oestrogen	5.85 and 6.27%	6.65 and 13.20%	0.1	<5%	1/50–1/2000
Progesterone	5.55 and 5.73%	9.12 and 10.76%	3.2	<5%	1/50–1/2000
5α-progesterone	5.32 and 6.36%	5.03 and 6.99%	2	<5%	1/50–1/8000

a Sample pools and individual samples were used for the parallelism tests depending on concentrations and volumes of the individual samples

### Data analysis

No analytical statistics were conducted due to the limited number of samples collected. The median value for each individual was calculated and compared with the different age and sex classes across the fPM, fEM and fAM results. Similarly, sex+age group median values were calculated and compared when possible. For the fAM assay validation adult males should have higher fAM values than juvenile males, whilst both male age classes should have higher fAM values than females. For the fEM and fPM assay validations, adult females should have higher fEM and fPM values than juvenile females, whilst both female age classes should have higher values than either male age class. Similarly, fPM and fEM of the pregnant female should be higher than all other female and male age classes. All values are presented as median and interquartile range (IQR). The IQR provides a robust measure of variability that is less sensitive to extreme values than the range or standard deviation.

## Results

### Faecal androgen metabolite measurements

The testosterone EIA only detected low fAM levels in both male and female individuals across all age groups. Additionally, testosterone metabolite concentrations in males (range: 0.05–0.98 ng/g dry weight (DW)) and females (range: 0.02–0.79 ng/g DW) exhibited significant overlap, with just one male sample exceeding the highest female sample (1.98 vs 0.79 ng/g DW). Therefore, the assay was deemed inadequate for detecting biologically relevant differences in fAM levels between sexes. In contrast, the epiandrosterone EIA revealed a substantial difference in fAM ranges between males (7.17–43.34 ng/g DW) and females (0.80–13.14 ng/g DW) in this study. Median epiandrosterone concentrations were notably higher in adult males compared to juvenile males, and both male age groups had higher median fAM levels than adult and juvenile females (Table 2).

**Table 2:** The median and range values for faecal testosterone and epiandrosterone metabolite concentrations between all age and sex classes

**ID**	**Assay**	**Median (ug/g DW)**	**Range (ug/g DW)**
Adult female 1	Testosterone Epiandrosterone	0.33 4.80	0.15–0.44 2.30–9.30
Adult female 2	Testosterone Epiandrosterone	0.06 8.72	0.05–0.15 6.44–13.14
Juvenile female 1	Testosterone Epiandrosterone	0.03 1.02	0.02–0.03 0.80–1.24
Juvenile female 2	Testosterone Epiandrosterone	0.72 1.52	0.56–0.79 0.80–1.95
Juvenile female 3	Testosterone Epiandrosterone	0.51 3.07	0.31–0.66 1.57–3.77
			
Adult male 1	Testosterone Epiandrosterone	0.58 21.47	0.43–1.98 19.86–43.34
Adult male 2	Testosterone Epiandrosterone	0.11 24.72	0.08–0.13 12.92–24.92
Adult male 3	Testosterone Epiandrosterone	0.38 19.85	0.10–0.71 12.07–24.94
Juvenile male 1	Testosterone Epiandrosterone	0.13 9.94	0.05–0.24 7.17–22.07
Juvenile male 2	Testosterone Epiandrosterone	0.11 14.45	0.08–0.29 7.37–21.24

### Faecal oestrogen metabolite measurements

Male fEM levels were at or below the detection limit of the oestrogen EIA, ranging from 0.01 to 0.02 ng/g DW. In contrast, female fEM levels were distinctly higher, ranging between 0.02 and 0.62 ng/g DW. Median fEM levels for individual adult females were notably higher than those of juvenile females (Table 3). Similarly, the fEM levels were considerably higher for the pregnant female sample than all other age and sex classes (Table 3).

**Table 3:** The median and range values for faecal oestrogen metabolite concentrations between adult and juvenile females as well as the pregnant female. All adult and juvenile male values were <0.02 and thus excluded from this table

**ID**	**Median (ug/g DW)**	**Range (ug/g DW)**
Adult female 1	0.42	0.42–0.62
Adult female 2	0.33	0.19–0.36
Juvenile female 1	0.12	0.03–0.48
Juvenile female 2	0.09	0.02–0.24
Juvenile female 3	0.07	0.04–0.14
Pregnant female	0.83	N/A

### Faecal progestagen metabolite measurements

Both progestagen EIAs effectively measured adequate fPM levels in Temminck’s pangolin. In both assays, fPM ranges were significantly higher in females (Progesterone EIA range: 1.51–12.64 ng/g DW; 5α-progesterone EIA range: 1.12–19.03 ng/g DW) compared to males (Progesterone EIA range: 0.95–2.42 ng/g DW; 5α-progesterone EIA range: 0.74–3.09 ng/g DW). Across both assays, median fPM levels for individual adult females were higher than those of juvenile females, and both adult and juvenile females had median fPM levels distinctly higher than all male study animals (Table 4). Finally, group-specific median fPM levels were higher in adult females compared to juvenile females, whilst both female groups had higher median values than adult and juvenile males. The pregnant female had an fPM level higher than all other age and sex classes (Table 4).

**Table 4:** The median and range values for faecal progesterone and 5α progesterone metabolite concentrations between all age and sex classes including the pregnant female

**ID**	**Assay**	**Median (ug/g DW)**	**Range (ug/g DW)**
Adult female 1	Progesterone	9.39	2.40–10.73
	5α Progesterone	9.60	3.08–13.92
Adult female 2	Progesterone	7.49	1.51–12.64
	5α Progesterone	9.19	2.05–19.03
Juvenile female 1	Progesterone	3.11	2.32–6.08
	5α Progesterone	4.69	3.34–9.49
Juvenile female 2	Progesterone	3.82	3.18–5.18
	5α Progesterone	4.07	3.11–4.28
Juvenile female 3	Progesterone	3.32	1.98–3.84
	5α Progesterone	1.45	1.12–2.56
Pregnant female	Progesterone	165.36	N/A
	5α Progesterone	233.44	N/A
Adult male 1	Progesterone	2.04	1.54–2.42
	5α Progesterone	1.98	0.94–1.98
Adult male 2	Progesterone	1.29	1.11–2.30
	5α Progesterone	1.56	1.55–3.09
Adult male 3	Progesterone	1.80	1.32–2.16
	5α Progesterone	1.58	1.26–1.59
Juvenile male 1	Progesterone	0.95	0.92–1.10
	5α Progesterone	0.94	0.74–0.95
Juvenile male 2	Progesterone	0.93	0.84–1.25
	5α Progesterone	1.14	0.94–1.50

## Discussion

This is the first study to validate EIAs for a reliable determination of gonadal endocrine patterns using faeces as a matrix in Temminck’s pangolin*.* The use of a biological validation process was sufficient to validate the respective EIAs. Reproductive activity can now be monitored in male and female Temminck’s pangolin via non-invasive endocrine monitoring.

The epiandrosterone EIA was successfully validated for measuring fAM concentrations in the Temminck’s pangolin. This finding confirms the applicability of this EIA for monitoring reproductive function in pangolins, as it has also been deemed suitable for quantifying fAMs in the Taiwanese pangolin (*Manis pentadactyla pentadactyla*; Arora *et al.,* 2020). Adult male Temminck’s pangolin had considerably higher fAM levels than juvenile males, highlighting the ability of the epiandrosterone EIA to discriminate between different maturation stages in males of this species. Similarly, the epiandrosterone EIA exhibited the capability to distinguish between male and female individuals across all age classes, revealing only a minimal overlap observed between juvenile males and adult females. The detection of such fAM differences has been observed and utilized as a biological validation tool in various mammalian species (Pineda-Galindo *et al.,* 2017; Scheun *et al.,* 2017), further affirming the reliability of employing this assay in Temminck’s pangolin.

The tested total oestrogen EIA was also successfully validated for quantifying fEMs in Temminck’s pangolin. As the assay can measure various oestrogens, such as estrone (E1), estradiol (E2) and estriol (E3) (Denver *et al.,* 2019), to name a few. As such, the antibody of the applied assay shows a recognizable cross-reactivity with a number of common oestrogens. However, Arora *et al.* (2020) suggested that estradiol-17β might be one of the primary oestrogens present in the faeces of pangolins. Whether this is the case for Temminck’s pangolin remains to be determined. Much like the discerning ability of fAM levels in males, the oestrogen EIA demonstrated its effectiveness in distinguishing between different age classes in females, affirming its ability to differentiate between various maturation stages in Temminck’s pangolin. Consistent with the outcomes of this study, previous research has also noted variations in female oestrogen levels associated with age (Kanematsu *et al.,* 2006; Musey *et al.,* 1987). Furthermore, fEM levels in male Temminck’s pangolin, when measured with the oestrogen EIA, were below the measurement threshold of the tested oestrogen EIA, indicating the ability of the EIA to differentiate between the sexes. Several studies have shown that males have lower oestrogen levels than their female counterparts (Barbosa-Moyano *et al.,* 2024; Gesquiere *et al.,* 2014). Therefore, the applicability of the oestrogen EIA in monitoring fEM concentrations in female Temminck’s pangolin was confirmed, demonstrated by its capability to distinguish not only between female age classes but also between male and females.

Both progestagen EIAs successfully discriminated between different age and sex classes; in addition to this, both EIAs also allowed for the determination of pregnancy. As a result, both progestogen EIAs are equally suitable to quantify fPMs in Temminck’s pangolin. Arora *et al.* (2020) reported comparable findings in the context of the Taiwanese pangolin, highlighting the prevalence of the 5α-configuration within the observed pregnane series. In this study, however, both assays demonstrated an ability to distinguish between adult females and their younger counterparts (i.e. able to differentiate between maturation stages). This ability is crucial when validating an EIA through biological means. Such differences in fPM levels between different age classes have been observed in previous research (Greenberg *et al.,* 2022) and supports the findings of this study. Finally, there were clear differences in fPM concentrations between females and males, with minimal overlap between juvenile females and adult males. This pattern has been observed in previous studies (Pineda-Galindo *et al.,* 2017) and support the validity of using either EIA to monitor fPM concentrations in Temminck’s pangolin.

Finally, although only one sample was available, both the fEM and fPM concentrations of the confirmed pregnant female Temminck’s pangolin were considerably higher than these metabolite concentrations in all other age and sex classes. An elevation in fPM and fEM concentrations during pregnancy has been observed in numerous species (Schwarzenberger *et al.,* 1996a; Nagl *et al.,* 2015; Scheun *et al.,* 2016) and used to validate the most appropriate EIAs for monitoring fEM and fPM levels in several species (Dehnhard *et al.,* 2008; Knott *et al.,* 2013; Mithileshwari *et al.,* 2016; Bleke *et al.,* 2021). This lends further support to our biological validations performed for both the fEM and fPM assays in Temminck’s pangolin.

## Conclusion

The EIAs validated in this study offer a powerful tool set for understanding the species’ reproductive patterns whilst also allowing for sex determination. In addition to this, information gathered can provide necessary parameters required to perform a robust Population Viability Analysis, which will be useful at present. Future research should focus on using these EIAs to determine female cyclicity and explore the connection between gonadal hormones and breeding behaviour. One limitation of the current study is the small sample size, a constraint due to the species’ conservation status as well as general ecology. Increasing the number of animals in future studies, along with long-term monitoring, would contribute significantly to our understanding of the reproductive patterns of this species.

## Data Availability

The data underlying this article are available in the article and in its online supplementary material.
